# Supplementary material for: Gender-based comparison of factors affecting regular exercise of patients with Non-Insulin Dependent Diabetes Mellitus (NIDDM) based on the 7th Korea National Health and Nutrition Examination Survey (KNHANES)
Source: PLoS One. 2021 Sep 30;16(9):e0257822. doi: 10.1371/journal.pone.0257822 (PMC8483364; doi:10.1371/journal.pone.0257822)
Supplement: Attachment — Submitted filename: Reviewers Responses to Questions.docx [file pone.0257822.s001.docx]

Reviewers' comments: Reviewer's Responses to Questions

**Comments to the Author**

1. Is the manuscript technically sound, and do the data support the conclusions?
The manuscript must describe a technically sound piece of scientific research with data that supports the conclusions. Experiments must have been conducted rigorously, with appropriate controls, replication, and sample sizes. The conclusions must be drawn appropriately based on the data presented.

Reviewer #1: Yes

2. Has the statistical analysis been performed appropriately and rigorously?

Reviewer #1: I Don't Know

Response: Our research was conducted in accordance with the analysis guidelines of the data provider (the Korea Centers for Disease Control and Prevention (KCDC)) and analyzed by statisticians.

3. Have the authors made all data underlying the findings in their manuscript fully available?

The [PLOS Data policy](http://www.plosone.org/static/policies.action#sharing) requires authors to make all data underlying the findings described in their manuscript fully available without restriction, with rare exception (please refer to the Data Availability Statement in the manuscript PDF file). The data should be provided as part of the manuscript or its supporting information, or deposited to a public repository. For example, in addition to summary statistics, the data points behind means, medians and variance measures should be available. If there are restrictions on publicly sharing data—e.g. participant privacy or use of data from a third party—those must be specified.

Reviewer #1: Yes

4. Is the manuscript presented in an intelligible fashion and written in standard English?

PLOS ONE does not copyedit accepted manuscripts, so the language in submitted articles must be clear, correct, and unambiguous. Any typographical or grammatical errors should be corrected at revision, so please note any specific errors here.

Reviewer #1: No

Response: We requested a specialized institution to edit English.

5. Review Comments to the Author

Please use the space provided to explain your answers to the questions above. You may also include additional comments for the author, including concerns about dual publication, research ethics, or publication ethics. (Please upload your review as an attachment if it exceeds 20,000 characters)

Reviewer #1: The authors investigate data from the KNHANES study with the aim to understand the gender factors that influence the regular exercise of patients with diabetes in Korea.

This kind of investigation is highly relevant for cardiovascular medicine, enabling personalized prevention, which is considered to dampen disease trajectories and thereby reduce healthcare costs in the future while increasing health span in the general population, but also in people with increased cardiovascular risk such as patients with diabetes. We know National investigations of this kind are important to better address local differences in lifestyle (and potentially genetic influences). Data from National studies are usually accepted better by policy makers and authorities. The study provides a wealth of results that can hopefully be used to implement prevention programmes.

The variables studied are clearly described.

The study does not discriminate between type 1 and type 2 diabetes, albeit both are different disease entities. This needs tob e amended.

Response: Thanks for pointing out. Diabetes was changed to Diabetes mellitus (DM), Type 1 to Insulin Dependent Diabetes Mellitus (IDDM), and Type 2 to Non-insulin Dependent Diabetes Mellitus (NIDDM).

In the abstract, phrasing needs tob e revised in order to better reflect that the analysis only provides associations, not causalities.

Response: that the analysis only provides associations.

Minor:
There are several grammatical mistakes and misleading phrases in the text. I would suggest to ask a native English speaker to proof-read the manuscript.
line 77-78: now: „… slightly intense exercise with the maximum heart rate of 50-70% for more than 150 minutes…“ suggest to change to „…moderately intense exercise with 50-70% of the maximal heart rate for more than 150 minutes…“
line 89: now: „…males tended to be engaged in physical activities that promote health than females…“ suggest to change into „…males tended to be engaged in physical activities that promote health more frequently than females…“
line 90: „…males were walking regularly than their female counterparts…“ suggest to change into „…males were walking regularly more often than their female counterparts…“

Response : We have corrected all of the above. (The color is displayed differently.)

Table 4: Please explain the abbreviation „mh_stress“

Response : “mh stress: When they responded that they feel a lot of stress during their daily lives, stressfulness” I added it as a sentence below the table

Thank you So much for checking in detail.

6. PLOS authors have the option to publish the peer review history of their article ([what does this mean?](https://journals.plos.org/plosone/s/editorial-and-peer-review-process#loc-peer-review-history)). If published, this will include your full peer review and any attached files.


If you choose “no”, your identity will remain anonymous but your review may still be made public.


**Do you want your identity to be public for this peer review?** For information about this choice, including consent withdrawal, please see our [Privacy Policy](https://www.plos.org/privacy-policy).

Reviewer #1: No
